# Birth injuries in late preterm and term neonates after instrumental delivery: a prospective study on short-term and developmental outcomes

**DOI:** 10.3389/fped.2025.1569513

**Published:** 2025-04-04

**Authors:** Fawzia Mohamed Elgharbawy, Sarfrazul Abedin, Rajai Albedaywi, Hoda Rahoma, Hakam Khatib, Abdelkhalk Khedr, Hakeem Nazdaf, Abdulla Asa'd Alshami, Lina Habboub, Mohammad A. A. Bayoumi, Einas E. Elmalik, Ashraf Gad

**Affiliations:** ^1^ Neonatal Intensive Care Unit (NICU), Al Wakra Hospital (AWH), Hamad Medical Corporation (HMC), Al Wakra, Qatar; ^2^ Neonatal Intensive Care Unit (NICU), Women’s Wellness and Research Center (WWRC), Hamad Medical Corporation (HMC), Doha, Qatar; ^3^ Department of Physiotherapy, Hamad Medical Corporation (HMC), Doha, Qatar; ^4^ Pediatric Department, Weill Cornell Medicine, Doha, Qatar

**Keywords:** operative vaginal delivery, instrumental vaginal delivery, birth injury, neonates, neurodevelopmental outcome, forceps delivery, vacuum delivery, birth trauma

## Abstract

**Background:**

Instrumental vaginal delivery is a common obstetric procedure with potential neonatal complications. This study evaluated birth injuries and neurodevelopmental outcomes in neonates born at ≥35 weeks’ gestation following instrumental vaginal delivery.

**Methods:**

A prospective observational cohort conducted over 2 years (2021–2022) at Al Wakra Hospital, Qatar to assess birth injuries, neonatal intensive care unit (NICU) admission rates, and neurodevelopmental outcomes at 18 months in neonates born via instrumental delivery.

**Results:**

The study included 390 neonates born via instrumental delivery, with 84 birth injuries occurring in 80 neonates (20.5%). Cephalohematoma was the most common injury (43/84, 51.2%), followed by subgaleal hemorrhage and bone fractures (9/84, 10.7%) and intracranial hemorrhage (2/84, 2.38%). One neonatal death was associated with the combined use of vacuum and forceps. Birth injuries were more frequent with the combined use of vacuum and forceps (aOR 4.1, *p* < 0.001), labor induction (aOR 2.2, *p* = 0.010), and showed a trend toward increased risk with >3 instrument applications (aOR 2.2, *p* = 0.067). NICU admission occurred in 25.3% of neonates, with significantly higher rates in those delivered using both vacuum and forceps (18.2% vs. 5.8%, *p* < 0.001). Neurodevelopmental assessment was performed on 289 infants, of whom 28 (9.68%) had abnormal outcomes. The communication domain was most affected (67.8%, 19/28), followed by personal-social (28.6%, 8/28), fine motor (21.4%, 6/28), problem-solving (17.9%, 5/28), and gross motor skills (10.7%, 3/28). One infant was affected in all domains. The combined use of vacuum and forceps was an independent risk factor for abnormal neurodevelopmental outcomes (aOR 3.87, *p* = 0.019).

**Conclusion:**

Instrumental vaginal delivery carries risks of birth injuries and neurodevelopmental challenges. Skilled application, careful assessment of indications, and long-term follow-up are essential to minimize complications and ensure optimal outcomes.

## Introduction

Instrumental or operative vaginal deliveries involve the use of a vacuum device or forceps to expedite the second stage of labor due to maternal or neonatal indications. When not performed correctly, these procedures can pose significant risks to both the mother and newborn (1, 2).

Studies have shown that the prevalence of instrumental vaginal deliveries ranges from 3% to 11% across different settings (2–5). However, the incidence can vary significantly, ranging from 1.5% to 15%. In the United Kingdom, instrumental vaginal delivery rates remain relatively stable at 10%–15%, though there has been a shift in the preferred instruments used (1, 3). According to the National Vital Statistics Report, forceps or vacuum-assisted vaginal delivery was used in 3.6% of births in the United States in 2010. Additionally, the Royal College of Obstetricians and Gynaecologists reported that instrumental deliveries accounted for approximately 11%–17.3% of births (6–8).

Instrumental vaginal delivery is indicated in cases of the prolonged second stage of labor, non-reassuring fetal heart rate tracing, or the need to shorten the second stage for maternal benefit. Both forceps and vacuum extraction have the potential to cause fetal and neonatal injury; however, vacuum-assisted delivery is usually associated with a lower incidence of maternal injury compared to forceps (9).

Although vacuum extraction is less likely to cause serious maternal injury, it has a lower success rate for achieving vaginal delivery compared to forceps-assisted delivery and is associated with a higher incidence of neonatal cephalohematoma. In contrast, forceps delivery is more often linked to facial and cranial injuries in neonates (10). In a study by Shimalis et al., vacuum-assisted delivery was linked to lower odds of complications (11).

To minimize both maternal and fetal risks, instrumental delivery should be indicated, and the operator must be well-trained and skilled in the proper application of the chosen instrument (12, 13). It is recommended that instrumental vaginal delivery be performed only from a low or outlet station, where the fetal head is well-engaged in the maternal pelvis to avoid complications such as birth trauma and failed extraction. Therefore, proper assessment of the fetal station and position is crucial to improve the outcome of the procedure (8, 14, 15).

Recent studies indicate a decreasing trend in instrumental vaginal deliveries, which is a growing concern worldwide as the rate of cesarean sections continues to rise (16, 17). Evaluating trends and indications for instrumental deliveries is critical for developing effective strategies to reduce cesarean section rates while mitigating associated risks and complications (18, 19).

While maternal complications related to instrumental delivery, such as postpartum hemorrhage and genital tract laceration, are well-documented (1, 20), evidence of neonatal morbidity following instrumental vaginal delivery remains inconsistent. A systematic review comparing vacuum extraction with forceps delivery found no significant differences in Apgar scores at one and five minutes and reported few serious neonatal injuries. However, vacuum extraction was associated with a higher incidence of cephalohematoma and retinal hemorrhage (11, 21).

Compared to full-term neonates, late preterm infants (gestational age 34–<37 weeks) are more vulnerable to complications during instrumental deliveries due to their physiological immaturity and increased risk of precipitous labor (22–24). These infants have a higher likelihood of birth trauma and related complications, often requiring neonatal intensive care unit (NICU) admission (25–29). Additionally, they experience greater morbidity and mortality rates than term infants [gestational age (GA) ≥37 weeks] due to their relative physiological and metabolic immaturity (30). A Swedish nationwide population-based study reported birth trauma rates ranging from 1.1 to 10 per 1,000 infants (31).

Despite previous research focusing on immediate outcomes linked to instrumental delivery such as maternal trauma and neonatal injuries, the long-term impact on neonatal development remains uncertain, creating a knowledge gap. Addressing this gap is critical for improving clinical practice and optimizing care for infants born by instrumental delivery.

This prospective observational study aimed to evaluate outcomes in neonates born ≥35 weeks via instrumental vaginal delivery, specifically assessing the incidence of birth injuries and NICU admissions. Additionally, we investigated the developmental outcomes of these infants at 18 months of age.

## Materials and methods

### Study design, population and setting

This prospective observational study was conducted at Al Wakra Hospital (AWH) between January 1, 2021, and December 31, 2022. We included infants born at ≥35 weeks’ gestation following instrumental delivery based on predefined inclusion and exclusion criteria, following written informed consent. AWH is a facility under Hamad Medical Corporation (HMC) located in Al Wakra City, Qatar. AWH provides comprehensive maternity services and is equipped with a Level II NICU.

During the study period, there were a total of 10,516 deliveries, with 2,498 admissions to the NICU. Among these, 6,200 were vaginal deliveries of infants at ≥35 weeks gestation. We collected data in real time from the electronic documentation system, Cerner software, and systematically recorded it in an Excel spreadsheet. Each patient was assigned a unique code to ensure anonymity, with these codes securely stored in a computerized system. A total of 390 neonates delivered via instrumental delivery were enrolled.

### Sample size calculation

The sample size of 688 neonates (344 in the instrumental group and 344 in the control group) was calculated to detect a difference of 2/100 between groups in birth injury rate, with 80% power and a 5% significance level. The standard normal deviate for a = Za = 1.9600 The standard normal deviate for B = Zß = 0.8416 Pooled proportio*n* = *P* = (q1*P1*) *+* *(q0*P0) A = ZaVP(1-P)(1/q1 + 1/q0) = 0.6296 B = ZßVP1(1-P1)(1/q1) + PO(1-PO)(1/q0) C = (P1-P0)2 Total group size = *N* = (A + B)2/C Continuity correction (added to N for Group 0) = CC = 1/(q1 * |P1-POl). Due to restrictions during the COVID-19 outbreak, no neurodevelopmental assessment was conducted for any of the control groups, and only 289 participants in the intervention group underwent assessment. Furthermore, the control group was excluded from the analysis to focus specifically on the outcomes of birth injury in the instrumental delivery group.

### Inclusion and exclusion criteria

Our study included cases of instrument-assisted vaginal delivery in cephalic presentation, singleton pregnancies, gestational ages between 35 and 41 + 6 weeks, and birth weights (BW) above 2,000 g.

Neonates with congenital anomalies, inborn errors of metabolism, non-cephalic presentations, cesarean deliveries, incomplete maternal data, and culture-proven early-onset sepsis were excluded.

### Maternal data

We collected the following data for mothers; age, parity, gestational age, use of labor induction, mode of delivery, the instrument used and type, number of applications, indication for instrument used, fetal distress, baseline fetal heart rate <100, reduced or increased variability/sinusoidal pattern, repetitive late or prolonged decelerations, and meconium-stained amniotic fluid (MSAF).

### Neonatal data

We collected neonatal data, including GA, BW, sex, Apgar scores at 1 and 5 min, need for positive pressure ventilation, birth injuries, NICU admission, and its indication, seizures within the first 24 h, birth asphyxia, highest level and duration of respiratory support, feeding, and sucking ability, antibiotic administration within the first 24 h, duration of antibiotic use, and length of NICU stay. Laboratory data included white blood cell count, lowest platelet count, and highest C-reactive protein (CRP) levels.

### Outcomes

In this study, the primary outcome was rates and types of birth injury, which included soft tissue injuries, bone fractures (skull, clavicle, and humerus), cephalohematoma, subgaleal hemorrhage, intracranial hemorrhage, and nerve palsies such as Erb's palsy and facial nerve palsy. Additionally, we examined NICU admission rates and neurodevelopmental outcomes at 18 months.

Neonatal depression is characterized by low Apgar scores (≤7 at 5 min), respiratory distress, hypotonia, acidosis, and the need for resuscitation not meeting the definition of birth asphyxia. Birth asphyxia was defined as a pathological condition of impaired gas exchange or inadequate blood supply, leading to persistent hypoxemia and hypercarbia occurring in temporal proximity to labor (peripartum) and delivery (intrapartum). Following international criteria, a neonate was diagnosed with an acute perinatal hypoxic-ischemic event if the following signs were present: an Apgar score <5 at 5 and 10 min, fetal umbilical artery pH <7.0 or base deficit ≥12 mmol/L (or both), brain injury on magnetic resonance imaging (MRI) or MR spectroscopy consistent with acute hypoxia-ischemia, and multisystem organ failure indicative of hypoxic-ischemic encephalopathy (32).

### Neurodevelopment outcome

Neurodevelopmental assessment was conducted by a pediatric physical therapist with expertise in developmental evaluation. The Ages and Stages Questionnaire, Third Edition (ASQ-3), was used for assessment at 18 months of age. The ASQ-3 consists of 30 items across five developmental domains (six items per domain): communication, gross motor skills, fine motor skills, problem-solving, and personal-social skills (23, 24, 30, 33).

According to the guidelines of the ASQ-3, a score of more than 2.0 standard deviations (SD) below the mean serves as the referral cut-off, indicating the need for further evaluation. A child was considered screen-positive if their score fell below the referral threshold in any of the five domains. Age-specific cut-off scores were applied according to the ASQ-3 manual.

### Ethical approval and consent to participate

Our study strictly adhered to the ethical principles of the Declaration of Helsinki, Good Clinical Practice guidelines, and the regulations set by the Ministry of Public Health in Qatar. Ethical approval was obtained from the Institutional Review Board and the Medical Research Center (MRC) at HMC, Qatar, under protocol number MRC-01-19-456. The study followed the STROBE guidelines to ensure transparency and accuracy in reporting.

Informed consent, available in both Arabic and English, was obtained from all participants using a pre-approved consent form authorized by the MRC at HMC, Doha, Qatar.

### Statistical considerations and data analysis

We collected anonymized data and inputted it into a standardized electronic database, tailored to accommodate the study's design and objectives. Descriptive statistics were employed to summarize all clinical data related to both the mother and neonate.

Results were presented as mean and SD for continuous, normally distributed data; median and interquartile range (IQR) for continuous, non-normally distributed data; and frequencies and percentages for categorical data. Key findings were depicted using suitable statistical graphs. Associations between qualitative variables were evaluated using either the chi-square (*χ*^2^) test or Fisher's Exact test, as appropriate. For quantitative variables, means among independent groups were compared using unpaired *t*-tests for normally distributed data and the Mann–Whitney *U* test for non-normally distributed data.

Univariate analyses were conducted for data exploration and descriptive analysis and to identify candidate variables for multivariable logistic regression analysis at *p* < 0.25. This liberal approach allows for the inclusion of variables that may not reach traditional statistical significance but could still be clinically relevant. Multivariate logistic regression analysis was done to determine risk factors associated with abnormal neurodevelopmental assessments in neonates delivered by instrumental delivery by determining adjusted odds ratios (ORs) and confidence intervals. Logistic regression was utilized to compute adjusted odds. All presented *p*-values were two-tailed, with a value less than 0.05 considered statistically significant. Statistical analyses were conducted using the STATA/BE18 software package.

## Results

**Figure 1:**
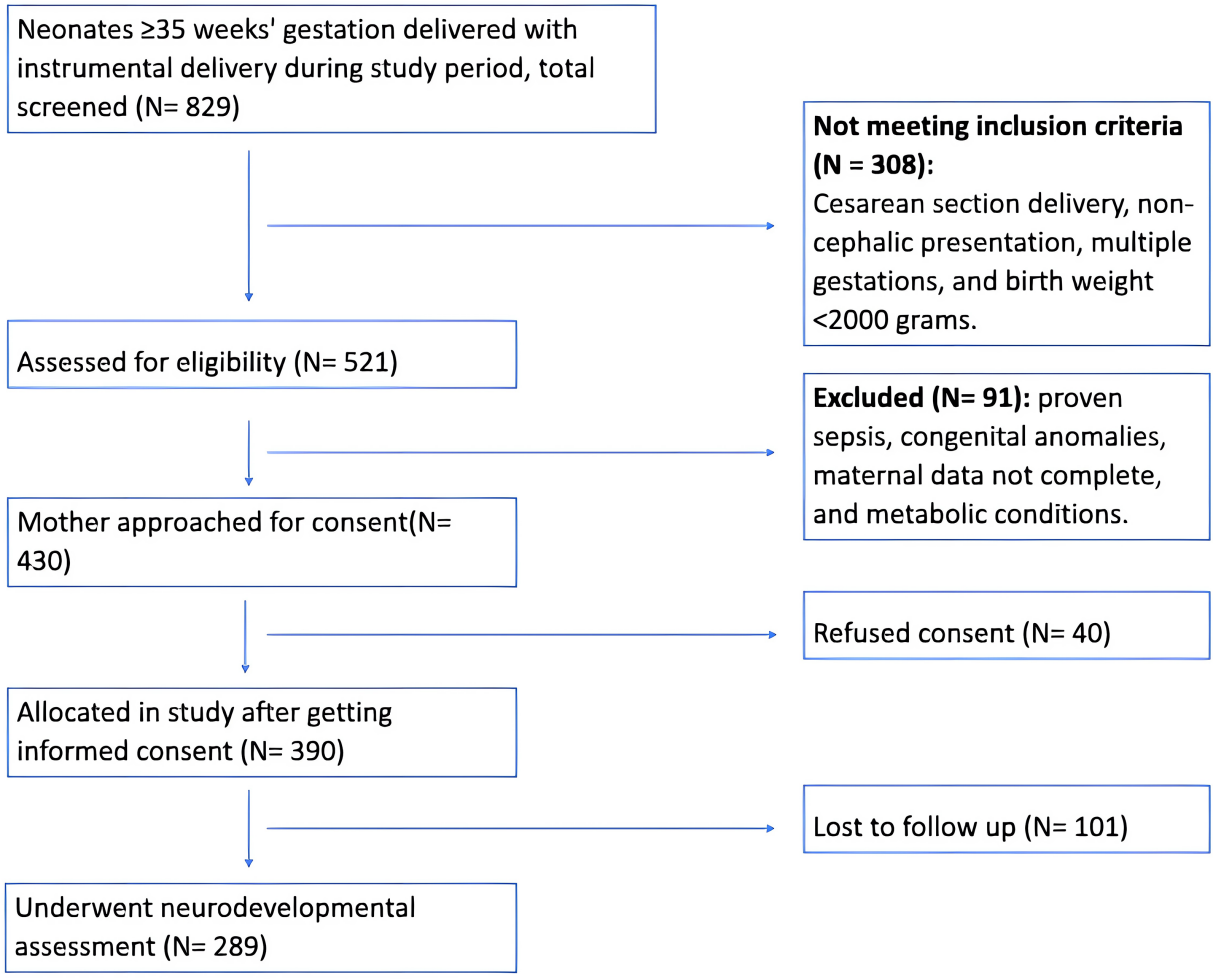
Study flow chart.

After screening 829 infants for the study eligibility, there were 521 eligible infants ≥35 weeks’ gestation born via instrumental vaginal delivery. After exclusions and consent refusals, a total of 390 infants were included in the study. Of these, 289 neonates underwent neurodevelopmental assessment, with the primary limitation being the impact of the COVID-19 pandemic during the study period. Figure 1 illustrates the study flow chart.

Table 1 highlights key characteristics of the neonates delivered via instrumental vaginal birth (*n* = 390). Significant differences were observed between the no-birth injury (*n* = 310) and birth injury (*n* = 80) groups. Labor induction was significantly higher in the birth injury group (65.0% vs. 42.3%, OR = 2.65, *p* < 0.001). Neonates with birth injuries had higher rates of 1 min Apgar <7 (12.5% vs. 4.2%, OR = 3.26, *p* = 0.005), 5 min Apgar <7 (2.5% vs. 0%, *p* = 0.005), and positive pressure ventilation (13.8% vs. 4.2%, OR = 3.64, *p* = 0.003). Neonatal depression was significantly more common in the birth injury group (10.1% vs. 1.6%, OR = 6.83, *p* = 0.001), as was poor sucking/hypoactivity (11.3% vs. 0.3%, OR = 39.04, *p* < 0.001). NICU admission rates were significantly higher in the birth injury group (25.4% vs. 7.1%, OR = 4.43, *p* < 0.001). Antibiotic use was more frequent (19.7% vs. 9.8%, OR = 2.26, *p* = 0.021) and of longer duration (1.67 vs. 0.72 days, *p* = 0.040) in the birth injury group. The highest CRP value was also significantly elevated in the birth injury group (20.0 vs. 4.0 mg/L, *p* = 0.032).

**Table 1:** Comparison of clinical and laboratory parameters between neonates with and without birth injuries delivered via instrumental vaginal delivery. *.*

Parameters	No birth injury (*N* = 310)	Birth injury (*N* = 80)	OR (95% CI)/MD (95% CI)	*P*-value
Mother age, years (mean ± SD)	29.98 ± 4.75	30.30 ± 4.96	0.328 (−0.886 to 1.54)	0.595
Multiparous [No. (%)]	136 (44.0%)	33 (41.3%)	0.893 (0.542 to 1.470)	0.657
Labor induction [No. (%)]	128 (42.3%)	52 (65.0%)	2.65 (1.582 to 4.406)	<0.001
Fetal distress [No. (%)]	236 (76.1%)	62 (77.5%)	1.080 (0.601 to 1.941)	0.797
Duration of second stage, min (mean ± SD)	101.27 ± 73.98	110.29 ± 56.65	−9.013 (−44.03 to 26.00)	0.610
MSAF [No. (%)]	48 (15.5%)	10 (12.5%)	0.780 (0.376 to 1.619)	0.504
Gestation age, weeks (mean ± SD)	39.03 ± 1.25	38.86 ± 1.18	0.170 (−0.137 to 0.477)	0.277
Male sex [No. (%)]	173 (55.8%)	46 (58.2%)	1.104 (0.669 to 1.820	0.699
Birth weight, grams (mean ± SD)	3,181.6 ± 481.8	3,254.8 ± 430.6	−73.21 (−189.54 to 43.11)	0.217
1 min Apgar <7 [No. (%)]	13 (4.2%)	10 (12.5%)	3.264 (1.375 to 7.748)	0.005
5 min Apgar <7 [No. (%)]	0 (0.0%)	2 (2.5%)	0.201 (0.1.65 to 0.245)	0.005
Positive pressure ventilation [No. (%)]	13 (4.2%)	11 (13.8%)	3.642 (1.565 to 8.475)	0.003
Neonatal depression [No. (%)]	5 (1.6%)	8 (10.1%)	6.828 (2.169 to 21.497)	0.001
LSCS [No. (%)]^a^	9 (2.9%)	9 (11.3%)	4.239 (1.624 to 11.066)	0.004
Seizure [No. (%)]	1 (0.3%)	2 (2.5%)	7.897 (0.707 to 88.218)	0.109
Birth asphyxia [No. (%)]	1 (0.3%)	1 (1.3%)	3.911 (0.242 to 63.225)	0.369
NICU admission [No. (%)]	33 (7.1%)	99 (25.4%)	4.432 (2.90 to 6.75)	<0.001
NICU stay duration, days (mean ± SD)	2.51 ± 1.46	3.06 ± 2.24	−0.545 (−1.37 to 0.281)	0.194
Respiratory distress [No. (%)]	39 (12.6%)	14 (17.5%)	1.474 (0.756 to 2.873)	0.252
Poor sucking/hypoactivity [No. (%)]	1 (0.3%)	9 (11.3%)	39.042 (4.868 to 313.149)	<0.001
Respiratory support duration, hours (mean ± SD)	13.55 ± 16.19	29.62 ± 47.34	−16.070 (−34.76 tto 2.621)	0.090
Any respiratory support [No. (%)]	33 (10.6%)	31 (16.3%)	1.629 (0.813 to 3.264)	0.166
Mechanical ventilation [No. (%)]	3 (30%)	31 (63.2%)	0.978 (0.218 to 4.383)	0.572
Antibiotic used [No. (%)]	27 (9.8%)	14 (19.7%)	2.256 (1.113 to 4.574)	0.021
Duration of antibiotic use, days (mean ± SD)	0.72 ± 1.49	1.67 ± 3.37	−0.948 (−1.852 to −0.43)	0.040
WBC, 10^3 ^/ul (mean ± SD)	16.15 ± 5.45	18.23 ± 8.33	−0.207 (−5.121 to 0.982)	0.181
Platelets, 10^3 ^/ul (mean ± SD)	259.06 ± 85.88	246.45 ± 87.77	12.61 (−27.85 to 53.09)	0.537
Highest CRP value, mg/L (mean ± SD)	4.0 ± 15.2	20.0 ± 44.5	−15.14 (−28.95 to −1.34)	0.032

OR, adjusted Odds Ratio; MD, Mean difference; mean ± SD, mean ± standard deviation; No. (%), number (percentage); 95% CI, 95% confidence interval; MSAF, Meconium-stained amniotic fluid; LSCS, lower segment cesarean section; NICU, neonatal intensive care unit; CPAP, continuous positive airway pressure; WBC, white blood cell; CRP, C-reactive protein.

Laboratory tests were performed as follows: WBC in 128 cases (91 no-injury, 37 injury group), platelet count in 119 cases (83 no-injury, 36 injury group), and CRP in 73 cases (56 no-injury, 17 injury group).

^a^ After failed instrumental vaginal delivery.

No significant differences were found in maternal age, parity, fetal distress, duration of the second stage of labor, meconium-stained amniotic fluid, gestational age, sex, birth weight, seizure rates, birth asphyxia, NICU stay duration, respiratory distress, or platelet counts.

**Figure 2:**
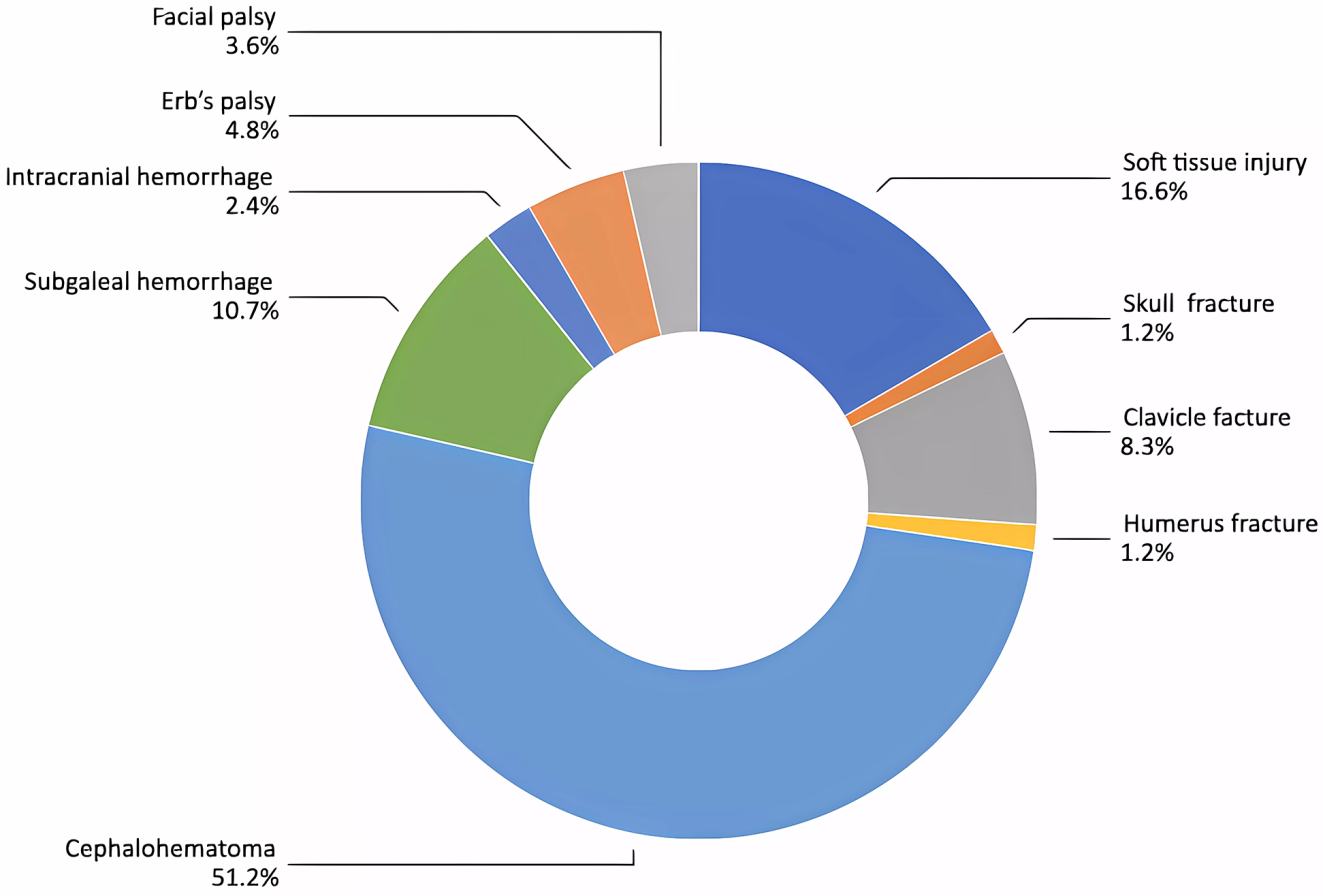
Birth injuries among neonates born by instrumental delivery.

Figure 2 illustrates the total birth injuries among neonates delivered using instruments, occurring in 80 out of 390 infants (20.5%). A total of 84 injuries were recorded among these 80 infants. The most common injury was cephalohematoma, accounting for 43 out of 84 injuries (51.2%). Subgaleal hemorrhage and bone fractures were observed in 9 out of 84 injuries (10.7%), while intracranial hemorrhage was reported in 2 out of 84 injuries (2.4%). One term infant, delivered using both vacuum and forceps assistance, developed subgaleal hemorrhage, HIE, and multiorgan failure, leading to death on day 5 of life.

Table 2 highlights the differences in instrumental delivery between neonates with birth injury and those without birth injury. The type of instrument used and the number of applications significantly differed between the no-birth injury and birth injury groups. Vacuum extraction was more common in the no-injury group (87.7% vs. 67.5%), while combined vacuum and forceps use was higher in the injury group (22.5% vs. 5.5%, *p* < 0.001). The injury group also had a higher median number of applications [2 (IQR 1–3) vs. 2 (IQR 1–2), *p* < 0.001]. Indications for instrumental delivery, such as fetal distress or prolonged second stage, did not differ significantly between groups (*p* = 0.215). These results highlight that instrument type and application frequency may influence birth injury risk, while indications for use do not.

**Table 2:** Differences in instrumental delivery between neonates with birth injury and those without birth injury.

Parameters	No birth injury (*n* = 310)	Birth injury (*N* = 80)	*X* ^2^ */Z*	*P*-value
Type of instrument				
Vacuum [No. (%)]	272 (87.7%)	54 (67.5%)		
Forceps [No. (%)]	21 (6.8%)	8 (10.0%)	24.523	<0.001
Both [No. (%)]	17 (5.5%)	18 (22.5%)		
Numbers of applications, median [IQR]	2 [1–2]	2 [1–3]	−3.552	<0.001
Indication				
Fetal distress [No. (%)]	214 (69.0%)	53 (66.3%)	4.467	0.215
Poor maternal effort [No. (%)]	47 (15.2%)	8 (10.0%)		
Prolonged second stage [No. (%)]	43 (13.9%)	18 (22.5%)		
Difficult extraction [No. (%)]	6 (1.9%)	1 (1.3%)		

IQR, interquartile range; *X*^2^/Z: *X*^2^ (Chi-square) for categorical data; Z (Z-score) for non-parametric continuous data.

Adjusted odds for birth injuries with various parameters were calculated using logistic regression analysis (Table 3). Among these parameters, “labor-induced” adjusted odds (95% CI) were 2.267 (1.267–4.056), “both vacuum & forceps used” adjusted odds (95% CI) 2.416 (1.034–5.644) and “number of instrument application” adjusted odds (95% CI) 1.296 (1.065–1.578) were independent risk factors for birth injury among instrument used group.

**Table 3:** Regression analysis of maternal and neonatal factors associated with birth injuries among neonates born by instrumental delivery.

Parameters	Adjusted ORs	95% confidence interval	*P*-value
Birth weight, grams	1.000	1.000–1.001	0.459
Low 1 min Apgar score^a^	1.969	0.718–5.395	0.188
Labor induction	2.2039	1.182–3.520	0.010
Type of instrument used			
Vacuum	1.0		
Forceps	1.893	0.763–4.702	0.69
Both vacuum & forceps	4.087	1.811–9.222	<0.001
Number of applications of the instrument			
1 application	1.0		
2–3 applications	1.420	0.763–4.702	0.247
>3 applications	2.263	0.945–5.422	0.067
Indications			
Prolonged second stage	1.0		
Poor maternal effort	0.591	0.217–1.609	0.303
Fetal distress	0.700	0.347–1.411	0.318
Difficult extraction	0.494	0.050–4.854	0.545

aOR, adjusted odds ratio.

1.0 = reference value.

^a^ Low Apgar score less than 7. The 5 min Apgar Score variable was removed due to an uninterpretable odds ratio, likely caused by computational issues resulting from the small number of patients in the affected category.

Table 4 highlights key factors associated with NICU admission in neonates delivered via instrumental vaginal delivery. Combined vacuum and forceps use (OR = 3.582, *p* < 0.001), prolonged second-stage labor (*p* = 0.017), labor induction (OR = 2.320, *p* < 0.001), and MSAF (OR = 2.024, *p* = 0.017) were significant risk factors. Instrument applications ≥3 increased NICU admission risk (OR = 2.199, *p* = 0.001), and a low Apgar score at one minute showed the strongest association (OR = 17.041, *p* < 0.001). Birth injury was also significantly linked to NICU admission (OR = 2.596, *p* < 0.001), emphasizing the potential risks of instrumental deliveries.

**Table 4:** Perinatal risk factors associated with neonatal intensive care admission among neonates born by instrumental delivery.

Parameters (*N* = 290)	No NICU admission (*N* = 291)	NICU admission (*N* = 99)	OR (95% CI) MD (95% CI)	*P*-value
Mother age, years (mean ± SD)	30.3 ± 5.0	29.9 ± 4.6	0.370 (−0.756 to 1.496)	0.518
Multiparous, [No. (%)]	141 (48.6%)	28 (28.3%)	0.417 (0.254 to 683)	<0.001
Labor induced, [No. (%)]	119 (40.9%)	61 (61.6%)	0.2.320 (1.545 to 3.704)	<0.001
MSAF, [No. (%)]	36 (12.4%)	22 (22.2%)	2.024 (1.124 to 3.645)	0.017
Duration of second stage, min (mean ± SD)	92.2 ± 58.5	131.3 ± 87.9	−39.1 (−71.131 to −7.037)	0.017
LSCS [No. (%)]^a^	12 (4.1%)	6 (6.1%)	1.500 (0.548 to 4.109)	0.414
Indication [No. (%)]				
Prolonged second stage	40 (13.7%)	20 (20.2%)	1.589 (0.878 to 2.875)	0.124
Poor maternal effort	45 (15.5%)	10 (10.1%)	0.614 (0.297 to 1.271)	0.185
Fetal distress	199 (68.4%)	68 (68.7%)	1.014 (0.620 to 1.658)	0.955
Difficult extraction	7 (2.4%)	0 (0.0%)	0.742 (0.699 to 0.787)	0.199
Instrument used				
Vacuum, [No. (%)]	250 (85.9%)	76 (76.8%)	0.542 (0.306 to 0.960)	0.034
Forceps, [No. (%)]	24 (8.2%)	5 (5.1%)	0.592 (0.219 to 1.595)	0.295
Both, [No. (%)]	17 (5.8%)	18 (18.2%)	3.582 (1.765 to 7.268)	<0.001
Instrument applications ≥3: [No. (%)]	70 (24.2%)	40 (40.8%)	2.199 (1.354 to 3.572)	0.001
Gestational age, weeks (mean ± SD)	39.0 ± 1.2	38.8 ± 1.3	0.159 (−0.126 to 0.444)	0.273
Male, [No. (%)]	164 (55.6%)	22 (55.6%)	0.960 (0.607 to 1.520)	0.863
Birth weight, gram (mean ± SD)	3,189 ± 416	3,216 ± 608	−26.303 (−134.410 to 81.803)	0.633
Apgar <7 at 1 min, [No. (%)]	4 (1.4%)	19 (19.2%)	17.041 (5.637 to 51.518)	<0.001
Apgar <7 at 5 min, [No. (%)]	0 (0.0%)	2 (2.0%)	0.250 (0.210 to 297)	0.064
Birth injury, [No. (%)]	47 (16.2%)	33 (33.3%)	2.596 (1.541 to 4.373)	<0.001

OR, adjusted odds ratio; MD, mean difference; mean ± SD, mean ± standard deviation; No. (%), number (percentage); 95% CI, 95% confidence interval; MSAF, meconium-stained amniotic fluid; LSCS, lower segment cesarean section.

^a^ After failed instrumental vaginal delivery.

### Neurodevelopmental outcome

**Figure 3:**
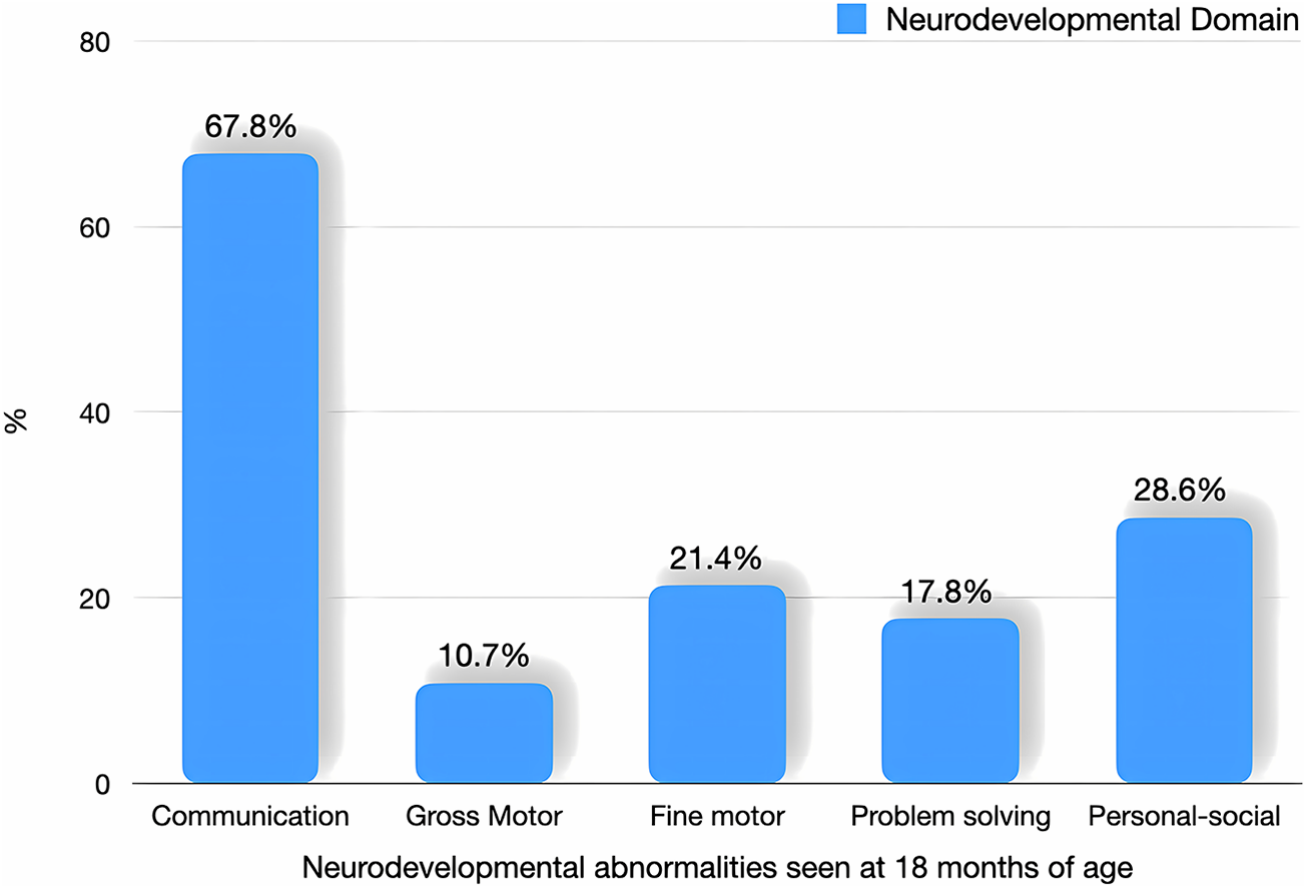
Neurodevelopmental outcomes among neonates delivered by instrumental delivery.

The neurodevelopmental assessment was done in 289/390 neonates (74.1%) using the Ages and Stages Questionnaire (third edition; ASQ-3) at the age of 18 months. Among them, 28 (9.68%) had abnormal ASQ outcomes. Among different developmental domains, communication was most affected by 19/28 neonates (67.8%); others gross motor skills 3/28 (10.7%), fine motor skills 6/28 (21.4%), problem-solving 5/28 (17.8%) and personal-social 8/28 neonates (28.6%). One infant developed global developmental delay with microcephaly and dystonic cerebral palsy. The number and categorization of neonates having abnormal neurodevelopmental outcomes following instrumental delivery, as assessed using the Ages and Stages Questionnaire (third edition; ASQ-3) were seen in Figure 3.

Table 5 presents the univariate and multivariate regression analysis of perinatal factors associated with abnormal neurodevelopmental assessment in neonates delivered via instrumental vaginal delivery. In the regression analysis, the use of both vacuum and forceps for delivery was significantly associated with abnormal ASQ outcomes (aOR = 3.874, *p* = 0.019). Lower gestational age showed a trend toward significance (aOR = 0.719, *p* = 0.061), while the male sex, which was initially significant in univariate analysis (OR = 2.954, *p* = 0.018), lost significance after adjustment (aOR = 2.334, *p* = 0.106). Poor sucking/hypoactivity also demonstrated a strong association with abnormal ASQ outcomes but did not reach statistical significance (aOR = 5.799, *p* = 0.085). Birth injury was not an independent variable in patients with abnormal ASQ outcomes. These findings suggest that combined instrumental delivery methods and lower gestational age may contribute to adverse neurodevelopmental outcomes in neonates.

**Table 5:** Maternal and neonatal risk factors associated with abnormal neurodevelopmental assessments in neonates delivered by instrumental delivery.

Parameters (*N* = 289)	Normal ASQ test (*N* = 261)	Abnormal ASQ test (*N* = 28)	OR (95% CI) MD (95% CI)	*P*-value	aOR (95% CI)	*P*-value
Birth injury, [No. (%)]	50 (19.2%)	6 (21.4%)	1.151 (0.443 to 2.987)	0.773		
Mother age, years (mean ± SD)	30.4 ± 4.9	29.2 ± 4.9	1.25 (−0.68 to 3.18)	0.932	0.940 (0.850 to 1.020)	0.127
Multiparous, [No. (%)]	118 (45.2%)	10 (37.0%)	0.713 (0.315 to 1.616)	0.416		
Labor induced, [No. (%)]	124 (47.5%)	10 (35.7%)	0.613 (0.27 to 1.38)	0.234	0.562 (0.783 to 1.447)	0.232
Indication [No. (%)]						
Prolonged second stage	43 (16.5%)	5 (17.9%)	1.0		1.0	
Poor maternal effort	32 (12.3%)	9 (32.1%)	2.419 (0.883 to 12.084)	0.144	3.266 (0.883 to 12.084)	0.076
Fetal distress	189 (69.3%)	14 (50%)	0.665 (0.227 to 1.947)	0.457		
Difficult extraction	5 (1.9%)	0 (0.0%)	0.000 (0.000 to .)^a^	0.999		
Meconium-stained amniotic fluid, [No. (%)]	38 (14.6%)	2 (7.14%)	0.451 (0.102 to 1.98)	0.280		
Instrument used						
Vacuum, [No. (%)]	223 (85.4%)	20 (71.4%)	1.0		1.0	
Forceps, [No. (%)]	18 (6.9%)	2 (7.14%)	1.239 (0.268 to 5.726)	0.784		
Both, [No. (%)]	20 (7.6%)	6 (21.4%)	3.345 (1.205 to 9.282)	0.020	3.874 (1.244 to 12.068)	0.019
Instrument applications ≥3: [No. (%)]	71 (27.5%)	8 (29.6%)	1.109 (0.465 to 2.647)	0.816		
Gestational age, weeks (mean ± SD)	39.1 ± 1.2	38.5 ± 1.1	0.720 (0.528 to 0.980)	0.037	0.719 (0.509 to 1.015)	0.061
Male, [No. (%)]	145 (55.6%)	22 (78.6%)	2.954 (1.159 to 7.525)	0.018	2.334 (0.836 to 6.513)	0.106
Birth weight, gram (mean ± SD)	3,195 ± 475	3,193 ± 515	1.815 (−185.5 to 189.5)	0.985		
Apgar <7 at 1 min, [No. (%)]	16 (6.1%)	1 (3.6%)	0.567 (0.072 to 1.085)	0.584		
Neonatal depression, [No. (%)]	7 (2.7%)	1 (3.57%)	1.333 (0.15 to 11.24)	0.791		
NICU admission, [No. (%)]	68 (26.1%)	10 (35.7%)	1.576 (0.69 to 3.58)	0.274		
NICU stay duration, days (mean ± SD)	3.04 ± 2.28	3.5 ± 3.2	−0.45 (−2.08 to 1.16)	0.578		
Respiratory distress, [No. (%)]	35 (13.4%)	5 (17.9%)	1.403 (0.508 to 3.93)	0.517		
Poor sucking/hypoactivity, [No. (%)]	5 (1.9%)	2 (7.4%)	4.09 (0.755 to 22.20)	0.133	5.799 (0.783 to 42.957)	0.085
Antibiotic used, [No. (%)]	28 (10.7%)	5 (17.9%)	1.809 (0.63 to 5.13)	0.260		

aOR, adjusted odds ratio; MD, mean difference; mean ± SD, mean ± standard deviation; No. (%), number (percentage); 95% CI, 95% confidence interval; ASQ-3, ages and stages questionnaire (third edition).

The reference group in the type of instrument used was “vacuum”, the indication of instrument used was “prolonged second stage”.

^a^ There were zero events in the difficult extraction group.

## Discussion

Our investigation was carried out to assess the birth injuries and developmental outcomes among neonates ≥35 weeks’ gestation following instrumental deliveries. The study included a total of 390 neonates, who were delivered with the assistance of instruments.

Among neonates in our study where instruments were used, 20.5% experienced birth injuries including one neonatal death and one case of cerebral palsy. This rate is slightly lower than the findings reported by Shimalis et al., who documented an overall complication rate of 37.2% in operative vaginal deliveries. Of those neonates, approximately one-quarter developed complications (11). In Canada, the rates of both maternal and severe neonatal birth injuries with instrumental vaginal delivery are increasing, especially with forceps delivery (34). Muraca et al. reported that the sequential vacuum and forceps application was associated with the largest increase in the rate of birth injury (ARR 1.53, 95% CI 1.03–2.27) (35).

In the past 3 decades, delivery by forceps has significantly declined to 1.1% of vaginal deliveries (5). Vacuum delivery has also declined but is more commonly used than forceps delivery. This may be because the vacuum is easy to apply compared to forceps. However, forceps use is more likely to result in a successful fetal extraction when compared to vacuum. This high incidence of forceps delivery might be related to the high rate of macrosomia and gestational diabetes mellitus in Qatar (36).

Hehir et al. showed that the rate of operative vaginal delivery increased significantly over 20 years, from epoch 1991–1995 to epoch 2006–2010, the incidence of instrumental delivery rose from 7.3% to 13.7%. The perinatal mortality rate decreased over time in instrumental deliveries (7.3/1,000 vs. 1.8/1,000). Despite a significant decline in forceps use from 68.2% to 32.9% in the same study, this rate remains significantly higher than the forceps use in our study, which was 7.43% (37). Our study, along with several others, consistently demonstrated a notable correlation between the types of instruments employed and neonatal outcomes. In our study, vacuum-assisted delivery accounted for 67.5% of birth injuries, a rate consistent with the 67% reported by Shimalis et al. However, neonates in our study did not exhibit as high a rate of low Apgar scores as observed in the latter study, where low Apgar scores were identified as the primary fetal complication, accounting for 45.7% of the major neonatal complications associated with vacuum-assisted delivery (11).

Evidence from observational data concerning instrumental deliveries suggests an association with neonatal injuries, emphasizing the importance of cautious practice and simulation-based training for obstetric healthcare professionals to achieve competency and minimize these risks (38). Labor induction, mode of delivery, type of instrument, and several instrument applications were identified as independent risk factors for birth injury. In comparison to obstetricians who favored forceps in the Abenhaim et al. study, obstetricians without a specific instrument preference exhibited a higher incidence of instrumental vaginal delivery. In addition, there were increased rates of cesarean sections and elevated episiotomy rates in non-operative vaginal deliveries. Infants delivered by obstetricians without a preference for a particular instrument were less likely to experience significant bruising but were more likely to develop a cephalohematoma (39). Major complications related to forceps-assisted delivery were reported in the literature including a 14.3% rate of NICU admission, a 3.2% incidence of birth trauma, and a 2.1% occurrence of stillbirths (39–41).

Admission to NICU occurred more frequently following instrumental vaginal birth compared to spontaneous deliveries (33, 42). We observed that approximately one-quarter of neonates born via instrumental delivery were admitted to the NICU. While several factors, such as primigravida mothers, labor induction, prolonged second stage, MSAF, and low Apgar scores, were associated with NICU admission, the use of both vacuum and forceps, instrument applications three or more times, and birth injuries were identified as independent risk factors.

When evaluated in the context of labor analgesia, instrumental delivery may independently contribute to increased NICU admissions and adverse neonatal outcomes (43). However, this relationship is not well understood and may be influenced by other factors, such as the combined effects of analgesia, which could underlie neonatal outcomes and NICU admissions (44). Moreover, NICU admission should not be considered the sole reliable indicator of neonatal morbidity. In the study conducted by Lamba et al. on 70 babies born by forceps delivery, 3 cases of stillbirth and 2 early neonatal deaths were documented. The elevated fetal mortality rate could potentially be attributed to delayed arrival at healthcare facilities and improper handling by untrained birth attendants before hospital admission. Additionally, out of the 70 neonates, 9 infants required admission to the NICU. Of those 9 NICU admissions, 7 were discharged within three days (45).

In our study, among all neonates assessed for neurodevelopmental outcomes, nearly 10% exhibited abnormal ASQ scores, falling below 2 SD, including one case of global developmental delay with dystonic cerebral palsy. Although birth injury as the findings suggest as an outcome was not an independent risk factor for abnormal neurodevelopmental outcomes, contributing factors such as lower GA, male gender, and the use of both vacuum and forceps were identified as significant risk factors for abnormal neurodevelopmental outcomes. A study from Sweden reported that children born by vacuum had a high rate of long-term neurodevelopmental impairment in both motor skills (OR 2.2, 95% CI 1.3–3.8) and perception (OR 1.7, 95% CI 1.002–2.9) compared to those born by spontaneous vaginal delivery (46).

Nevertheless, a comprehensive population-based study conducted over the long term compared the developmental outcomes of children born via normal vaginal delivery vs. instrumental delivery. The study utilized the National Assessment Program—Literacy and Numeracy (NAPLAN) scores at eight years old as an indicator of school performance, serving as a proxy for neurodevelopmental assessment. The findings did not provide any evidence to suggest that the use of instrumental delivery is linked to increased neurodevelopmental complications at the age of eight years (47).

### Strengths and limitations

We recognize several strengths in our research methodology. The prospective design and the large number ensure the accuracy of data collection for the majority of variables under investigation, as these are systematically recorded and reported on an ongoing basis. One significant advantage of this study is its inclusion of a comprehensive and diverse patient population, encompassing both nulliparous and multigravida mothers with low-risk singleton pregnancies. This wide-ranging sample enhances the generalizability of the study's findings to a broader population. The methodology employed, along with a rigorous statistical approach that categorizes patients into specific groups, enables a thorough comprehension of the effects of various analgesic methods on neonatal outcomes. Contrary to other studies, we focused on studying the relationship between instrumental delivery and the administration, quantity, and duration of antibiotics. Furthermore, conducting the study in a controlled environment, ensures uniformity in labor and delivery management practices across the study participants, thus minimizing potential confounding variables. Additionally, all instrumental deliveries were conducted by either an obstetrician or an obstetric resident under supervision, ensuring consistency and adherence to professional standards.

Due to our study being conducted at a single centre, there is a possibility of selection bias and reverse causality, particularly as it predominantly involves low-risk pregnancies, reflecting the available data. Furthermore, we did not incorporate data regarding women who underwent cesarean deliveries, individuals with intricate prenatal histories, or those with notable deviations in birth weight. Not all neonates born via instrumental vaginal delivery underwent developmental assessment at 18 months due to COVID-19 pandemic regulations. This limitation highlights the need for larger studies to validate these findings.

## Conclusion

Our study revealed adverse outcomes associated with instrument-assisted deliveries. We observed a heightened incidence of birth injuries and neonatal admissions to the NICU. Factors such as the type of combination of instruments used, labor induction, and the number of instrument applications correlated with birth injury complications arising from instrument-assisted delivery.

Moreover, certain factors including lower GA, male gender, and the utilization of both vacuum extraction and forceps were identified as risk factors for abnormal neurodevelopmental outcomes. These findings offer valuable insights for obstetric practitioners, enabling them to better comprehend the potential adverse effects following instrumental deliveries. Equipped with this understanding, clinicians can approach such situations with heightened awareness and discretion, thereby avoiding unnecessary interventions or undue alarm during labor, particularly when abnormalities are detected. Preventing operative vaginal birth and its subsequent complications through training, simulation, and maintaining competency should be a safety institutional goal in women's hospitals and should be checked and measured frequently. A thorough assessment of clinical circumstances is essential to identify indications and contraindications for instrument application which should be performed by the best available hands.

Furthermore, researchers can utilize our study as a foundation for conducting more comprehensive research, incorporating additional variables and integrating qualitative data for a deeper understanding. Long-term follow-up is recommended in those born with instrumental delivery for early detection and intervention of neurodevelopmental concerns.

## Data Availability

The raw data supporting the conclusions of this article will be made available by the authors, without undue reservation.
